# Is xylitol effective in the prevention of dental caries? A systematic review

**DOI:** 10.4317/jced.62008

**Published:** 2024-10-01

**Authors:** Bárbara Ortiz-Sáez, Miriam Aguilella-Traver, Caridad Hernández-Pando, Eva María Martínez-Salmerón, José Emilio Muñoz-Barrio, Gerardo Gómez-Moreno

**Affiliations:** 1 Master of Clinical Orthodontics and Maxillary Orthopedics, Catholic University of Ávila (UCAV), Odontomaster, Ávila, Spain; 2 Department of Dermatology, Stomatology, Radiology, and Physical Medicine, Faculty of Dentistry, University of Murcia, Murcia, Spain; 3 Department of Stomatology, Medically Compromised Patients, Faculty of Dentistry, University of Granada, 18071 Granada, Spain

## Abstract

**Background:**

Xylitol is a sugar alcohol increasingly used in dentistry as a preventative measure against dental caries. The objective of this systematic review was to assess xylitol’s efficacy in caries prevention through the reduction of the most commonly associated bacteria: Streptococcus mutans.

**Material and Methods:**

This systematic review followed PRISMA guidelines. A literature search was conducted in PubMed, Cochrane and Google Scholar databases. The search algorithm included the following key words: xylitol, dental caries, tooth demineralization, Streptococcus mutans, and prevention. The CASPe tool was used to assess risk of bias in the articles reviewed.

**Results:**

After the search and selection processes, nine clinical trials (some of them placebo-controlled) in humans were included in the review. The objective proposed – to assess the efficacy of xylitol in caries prevention – was not fulfilled conclusively in all the works. Little heterogeneity was observed among the trials, as the study groups, evaluation periods, daily doses of xylitol etc. varied considerably between the works.

**Conclusions:**

According to the present findings, the preventative effect of xylitol against dental caries cannot be confirmed. The results also highlight the need for further research with standardized protocols.

** Key words:**Xylitol, dental caries, dental demineralization, Streptococcus mutans, prevention.

## Introduction

Caries is considered one of the most widespread and established dental diseases in both children and adults. It is a multifactorial and polymicrobial disease that constitutes a public health problem all over the world (1,2). It is produced as the result of an imbalance (dysbiosis) between host, microorganisms, and environmental conditions (food), the main cause being the intake of sugar (carbohydrates). As cariogenic bacteria metabolize fermenTable carbohydrates, they produce acids that demineralize the teeth by dissolving the calcium and phosphate content of tooth enamel and dentin. Providing the predisposing factors are regulated, caries is a manageable and prevenTable disease (3,4). At present, caries management focuses on reducing sugar consumption through a range of preventative strategies. This includes the search for alternatives to sugar: artificial sweeteners or non-calorific sweeteners, such as sugar alcohols that are non-fermenTable by the bacteria most commonly involved the etiology of dental caries (1,2,4-6), these being *Streptococcus mutans* and *Lactobacillus* (2,7).

Xylitol, a sugar alcohol (polyol) with five carbon atoms, is a white-colored crystalline carbohydrate of natural origin, known for over a century. Its natural form occurs in fruits, berries, and vegetables and it has been studied extensively over the last 40 years because of its effect on dental caries. It can be produced artificially from vegetable materials rich in xylene, such as beech wood and birch (3,5,6,8). Xylitol was discovered by German chemist Emil Fisher *et al*. in 1891 (9) and its efficacy in reducing dental plaque has been investigated since the 1970s. In 1986, the U.S. Food and Drug Administration (FDA), declared xylitol safe for human consumption. Since then, it has been used in foods, pharmaceutical products, and in oral healthcare products in many countries (1,4-7,9). At present, it is manufactured and distributed in diverse forms, such as chewing gum, sweets, snacks, Tablets, etc. (1,9).

Xylitol is the sweetest of the sugar alcohols. It is characterized by having the same sweetness and volume as saccharine but with a third fewer calories and without requiring insulin for its metabolism, which contributes to its insulinemic properties and low glycemic index (2).

It has been shown that xylitol is a valuable agent in dental caries prevention because it is not an attractive substrate for the bacteria that make up oral biofilm. Its consumption increases saliva flow and reduces *Streptococcus mutans* (SM) levels by altering its processes of energy production, leading to an unusable energy cycle and cell death. At the same time, xylitol reduces the acidogenic potential and adhesion of these microorganisms to the tooth surfaces by increasing pH, which impedes enamel demineralization. Its main characteristic is that xylitol is practically non-fermenTable by oral bacteria (2,5,6).

The safety of xylitol has been extensively investigated. While most research has reported few secondary effects, such effects are produced only after high intakes of xylitol, as high as 50 g per day, four or five times the recommended dose. These include abdominal pain and diarrhea. But at the recommended dose of 6 g per day, xylitol is considered completely safe (1,5).

The objective of this systematic review was to determine the efficacy of xylitol in the prevention of dental caries through reducing *Streptococcus mutans*, the bacteria most commonly associated with caries.

## Material and Methods

This systematic review was conducted to fulfill PRISMA® 2020 (Preferred Reporting Items for Systematic Reviews and Meta-Analyses) criteria (10). It was registered in PROSPERO (International prospective register of systematic reviews), registration number CRD42024538588.

The objective of the review was to answer the following PICO question (11): (*P* = patient/population/problem; I = intervention; C = comparison; O = outcome): ¿Is xylitol a preventative agent against dental caries? P: Healthy adults and children, with or without fixed or removeable orthodontic devices/prostheses; I: treated with xylitol; C: Compared with a group or population not consuming xylitol; O: Efficacy of xylitol in reducing *Streptococcus mutans* levels.

The review included clinical trials in humans published in English or Spanish conducted over a 10-year period between January 2013 and December 2023.

An electronic search was made in the PubMed, Cochrane and Google Scholar databases using following MeSH (Medical Subjects Headings) terms and Boolean operators: xylitol, dental caries, tooth demineralization, *Streptococcus mutans*, and prevention. Combinations of these (MeSH) key terms were used: (“xylitol” [MeSH terms] OR “xylitol” [All fields]) AND (“dental caries” [MeSH terms] OR “dental” [All fields] AND “caries” [All fields] OR “dental caries” [All fields]) AND (“tooth demineralization” [MeSH terms] OR “tooth” [All fields] AND “demineralization” [All fields] OR “tooth demineralization” [All fields]) AND (“*Streptococcus mutans*” [MeSH terms] OR “Streptococcus” [All fields] AND “mutans” [All fields] OR “*Streptococcus mutans*” [All fields]) AND (“prevention” [MeSH terms] OR “prevention” [All fields]).

An additional manual search was made for any other articles that could be of relevance. All the articles selected for review fulfilled the following inclusion criteria: clinical trials in humans in good health; trials aiming to determine the preventative effect of xylitol against dental caries, regardless of age or the form of xylitol administration (chewing gum, sweets, mouthwashes, etc.). Exclusion criteria were: bibliographic reviews, systematic reviews, metanalyses, books or chapters of books, studies in which xylitol was not administered to subjects, animal or *in vitro* studies, incomplete studies lacking one or more of the established parts of a scientific study, articles whose main objective was not to analyze the effect of xylitol on caries.

Risk of bias assessment was carried out using the Critical Appraisal Skills Program CASPe, applying the following criteria: presence of a specific topic, preventative effect of xylitol against dental caries, relevance of the methods used to answer PICO question, relation with the study’s objective, and usefulness of the results, considering the reproducibility of the trial.

## Results

The initial electronic database search identified 395 articles. After eliminating duplicates, 17 further articles were discarded for being unobtainable, leaving the remaining articles (n=278) to the selection process. Applying the inclusion/exclusion criteria (studies conducted in humans, clinical trials, efficacy of xylitol for caries prevention) excluded a further 271, while several other works involved combinations of substances including xylitol, or compared xylitol with some other substance. Two articles that fulfilled the eligibility criteria were obtained from other sources or from cross-referencing.

**Figure 1:**
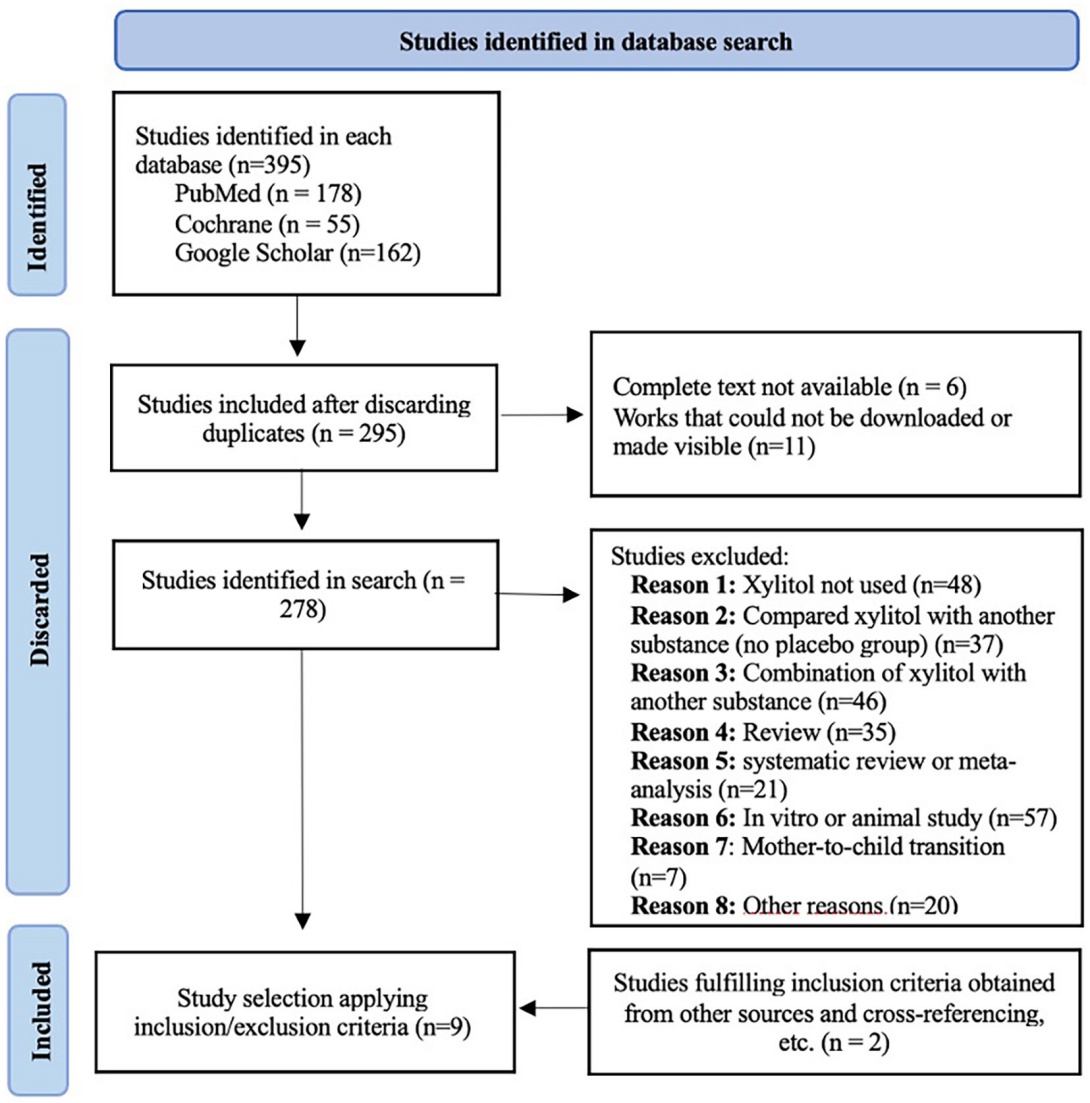
Flow diagram shows the study selection process (fulfilling PRISMA 2020 declaration) and number of articles selected for review (10).

**Figure 2:**
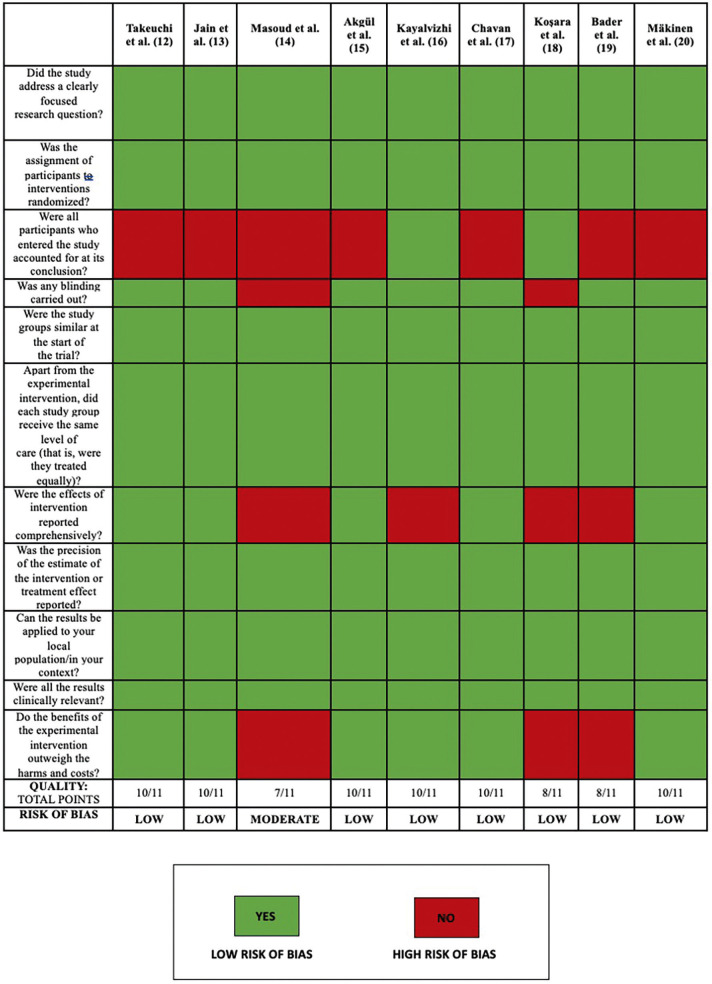
Assessment of study methods and risk of bias using CASPe checklist.

Nine clinical trials were finally included for review (Fig. 1), considering the following variables: study group, age, sex, form and pattern of xylitol administration, xylitol dosage, and study type ( Table 1). Critical reading of the articles applied CASPe criteria. All the studies were classified as being at low risk of bias, with the exception of one work considered at moderate risk of bias (Fig. 2).

The selected articles consisted of randomized controlled clinical trials, some with placebos, and prospective clinical trials. They included a total of 1,513 patients (including treated and control subjects), with ages ranging from 6 months to 80 years. Not much heterogeneity was found across the nine studies, as study groups, evaluation periods, and daily dosages of xylitol varied considerably.

Takeuchi *et al*. (12) evaluated the effect of xylitol chewing gum in order to determine whether short-term use reduced the total count of saliva bacteria. The trial included 76 patients, all men aged over 20 years, who were monitored for two days. The subjects were randomly divided into two groups: 39 chewed xylitol gum seven times a day for 5 minutes, while the control group (n=37) were not administered any gum. In this way, the test group received a dosage of 6.78 g xylitol per day. Stimulated saliva samples were collected at baseline and follow-up and their total saliva bacteria composition was evaluated with 16S rRNA gene sequencing. The total saliva bacteria count was measured by means of a quantitative real time PCR system (qPCR). Six subjects left the study and so were excluded from analysis, leaving 70 participants (34 test group and 36 control subjects) included in analysis. No statistically significant differences were found between the groups for the parameters analyzed in baseline samples. But in follow-up analyses the control group exhibited a significantly lower bacteria count than the control group. At the same time, no significant differences were found between the groups in terms of general bacterial composition between baseline and follow-up analyses.

Jain *et al*. (13) set out to compare the efficacy of xylitol chewing gum with a combination of IgY (immunoglobulin Y) and chewable xylitol Tablets for reducing SM in children. Children (n=120) of both sexes, aged between 6 and 12 years were included in the trial. The subjects selected presented SM saliva counts of ≤ 105 colony forming units and all had at least one tooth with caries, either lost or in treatment. They were divided randomly into three groups of 40: Group 1 chewed two pieces of xylitol chewing gum twice a day for 5 minutes; Group 2 consumed one chewable xylitol Tablet with IgY twice a day; and Group 3 (control group) were not administered xylitol in any form. Both test groups received approximately 1.6 g xylitol per day. The trial duration was 15 days and six subjects were excluded. Saliva samples were collected at baseline, after 15 days and 1, 2, and 3 months later. The samples were inoculated in mitis salivarius-bacitracin agar with potassium tellurite medium and the number of SM colony forming units (CFU) were counted. The data obtained underwent statistical analysis, finding significant differences in the numbers of CFUs between the groups, so that a higher number was observed in the control group than in Group 2 (IgY and xylitol).

Masoud *et al*. (14) aimed to evaluate the efficacy of 6 g xylitol per day on SM counts in plaque and saliva. The study included 41 subjects undergoing orthodontic treatment, of both sexes, aged between 12 and 30 years. They were divided randomly into three groups: Group 1 (n=13) received six pieces of xylitol chewing gum per day; Group 2 (n=13) 12 chewable soluble xylitol Tablets per day; and Group 3 (control group n=12) were not administered xylitol. Clinical examinations and saliva and plaque sample collections were performed at the start of the study and after 3, 6, and 12 months. Any patients who failed to comply with the regime were excluded. Plaque scores and bacteria counts were used to assess the efficacy of xylitol in caries reduction. No statistically significant differences were found between the three groups in plaque scores, or SM counts in plaque or saliva, at any of the study times.

Akgül *et al*. (15) investigated the effect of xylitol consumption in the short term on pro-inflammatory cytokines and SM counts using the enzyme-linked immunosorbent assay (ELISA) and qPCR. The trial included 154 participants of both sexes aged between 18 and 65 years, assigned to a xylitol group or a control group. Both groups chewed two pieces of gum for at least 10 minutes three times per day. The test group received 5.4 g xylitol per day. During the second week of the study, seven participants (three control group and four test group) were excluded due to antibiotic use. Intraoral dental examinations were carried out, saliva samples were collected with swabs, and gingival indices and plaque and microbiological levels were evaluated. In the xylitol group, gingival index and plaque values were significantly lower after three weeks than at baseline (*p*<0.001 and *p*<0.05, respectively), as well as the concentration of cytokines in saliva. SM was reduced by approximately five times through the use of xylitol over the three weeks with significant difference in comparison with baseline counts.

Kayalvizhi *et al*. (16) evaluated the effect of xylitol tooth wipes on SM in babies. Forty-four children of both sexes, aged between 19 and 35 months were selected to take part in the trial. They were divided into two groups randomly, one group treated with xylitol wipes (2.6 g per day) and the other with placebo wipes. The infants’ teeth were cleaned using the wipes twice a day for 14 days. Saliva samples were collected at the beginning and end of the study period and cultivated in mitis salivarius-bacitracin saccharose agar and bacitracin for SM, evaluating colony forming units per milliliter. No significant differences in the reduction of SM were found between the groups, although some reduction was observed in the xylitol group. Data were compared between baseline and the end-of-study evaluation, finding reductions in SM in both groups but without statistical significance.

Chavan *et al*. (17) evaluated the effect of a xylitol chewing gum, an herb-based chewing gum, and a placebo chewing gum on SM in saliva over a 21-day period. The study included 72 school students of both sexes aged 12-15 years (average age 14 years), with at least one caries and a SM saliva count of ≥105 UFC/ml of saliva. The teenagers were divided randomly into three groups: Group 1 were administered herb-based chewing gum; Group 2 1.28 g xylitol chewing gum; Group 3 placebo chewing gum. One piece of chewing gum was consumed four times per day, chewing for 10 minutes. Some subjects did not follow the regime adequately, mainly in the placebo group. Non-stimulated saliva samples were collected at the beginning and end of the study period. The chewing gum sweetened with 100% xylitol produced a statistically significant reduction in SM CFUs in saliva at the end of the 21-day study period. No significant reductions occurred with the herb-based gum or the placebo.

Kosara *et al*. (18) set out to determine whether toothbrushes impregnated with xylitol affected the periodontal condition and microbial flora in patients with poor oral hygiene wearing fixed orthodontic apparatus. All patients wore conventional metal orthodontic apparatus, and presented no indications for dental extractions. Forty-four patients participated in the trial (22 males and 22 females) aged between 12 and 18 years (mean age 14.38 +/- 1.96 years). All patients presented Quigley Hein (modified by Turesky) plaque index scores of 1.5 or over. They were divided randomly into two groups: 22 patients used toothbrushes impregnated with xylitol (test group) and 22 used toothbrushes without xylitol (control group). They were asked to use a new tooth brush every day for 12 weeks, brushing twice a day for 2 minutes. In this way, test patients were exposed to approximately 0.02 g xylitol at each brushing. Clinical periodontal parameters were recorded and saliva samples taken at three study times: before starting to use the toothbrushes (T0), four weeks later (T1), and 3 months later (T2), when periodontal health and changes to microflora were evaluated. Total bacteria levels were calculated by totaling SM and *Lactobacillus* numbers; the results were expressed as CFUs per milliliter. In the xylitol group, total bacteria decreased significantly from T1 to T2, with statistically significant difference for SM and for the total bacteria counts in both the xylitol and control group between T0 and T2. Comparisons between groups for all the microbial parameters did not exhibit significant differences at any of the study times.

Bader *et al*. (19) tested the efficacy of xylitol Tablets in reducing caries in high-risk adults. The trial included 691 patients of both sexes aged between 21 and 80 years (average age 47 years) with at least one caries and at least 12 teeth. They were divided randomly into two groups: a xylitol group of 344 subjects and a placebo group. They were asked to consume five Tablets containing 1 g xylitol per day for 33 months. Clinical examinations were carried out at the start of the study period and at 12, 24, and 33 months. Over the study period, 27 patients were excluded from the final analysis due to incomplete data. The trial did not find any statistically significant reduction in the incidence of caries over the 33 months, at the first analysis, or the second. The xylitol Tablets reduced the increase in caries by 11%, which represents a third of the dental surface per year, without statistical significance. There were no indications of a dose-response effect.

Mäkinen *et al*. (20) set out to explore the viability of topical applications of xylitol as a potential complementary measure in caries prevention in babies aged between 6 and 8 months and to determine its effects. Initially a total of 271 babies belonging to 266 families began the trial. 133 infants were assigned to the xylitol group applied by means of cotton swabs or toothbrushes. Xylitol was applied in the form of a 45% aqueous solution, instructing the parents to rub the surfaces of all the deciduous teeth present using the cotton swab or a small toothbrush. The treatment (cleaning with xylitol) continued for approximately 26-28 months, the quantity of xylitol applied being 13.5 g per day. Families who failed to follow the program adequately were excluded from the final analysis. Although caries was usually registered at the age of 7 years, caries data was also calculated at younger ages, showing significant reductions (*p* < 0.001) in the incidence of enamel and dentin caries in the xylitol group compared with the control group at all time intervals. In the test group, oral SM counts decreased significantly (*p* < 0.001).

## Discussion

Caries is an infectious and multifactorial disease caused by specific bacteria that adhere to teeth, in particular *Streptococcus mutans* (SM), which metabolizes sugars to produce acid that over time demineralizes the dental structure. Caries prevention is based on the search for substances that reduce or eliminate SM. For this reason, xylitol has been a widely researched topic in the field of preventative dentistry and numerous researchers have chosen to implement trials aimed at assessing the effects of xylitol. In particular, they have chosen to test xylitol-sweetened chewing gum, as mastication stimulates saliva that can neutralize or raise the pH of plaque and improve the elimination of fermenTable carbohydrates, through the mechanical action of chewing.

Takeuchi *et al*. (12) found significant differences in SM counts between test group and control subjects. The test group was given xylitol chewing gum, while the control group was not given any gum. It is known that mastication produces a mechanical cleaning action and that the number of bacteria is related to the amount of plaque on dental surfaces. So, these positive findings could be partly due to the effect of chewing, as it clears part of the biofilm. In any case, the authors themselves recognize the trial’s limitations, including the brief intervention of only two days. Similar limitations were observed in other articles reviewed here (13,14,20).

Jaeein *et al*. (13) obtained results whereby the xylitol group underwent a significant reduction in SM in comparison with the control group. The authors state that they preferred a control group “without chewing gum,” rather than a placebo gum, which could cause variations in SM levels and also has an unpleasant flavor, which could prove an obstacle to children using the gum with equal frequency and duration. Also, because – again – the mechanical cleaning action of chewing could be a source of bias. In addition, the children were asked to brush twice a day and to limit their consumption of sugar, a situation that could have skewed the results positively.

It should be noted that these results concur with those obtained by Mäkinen *et al*. (20), who found constantly lower bacteria counts in the test group that had received xylitol administered topically. Although the authors obtained the results they expected, they were aware of the high number of families who did not carry out the daily routine the trial imposed. Moreover, some families refused to administer xylitol but were happy to participate as control subjects. The participants generally responded badly to healthcare guidance, so, as the authors themselves point out, it is possible that the parents who gave their consent to participate in the xylitol group had greater awareness of healthcare practices and oral hygiene (toothbrushing) in general.

Masoud *et al*. (14) administered xylitol in two forms, as chewing gum and sweets. The control group were not provided with any placebo. Like most of the trials outlined above, the results revealed a constant reduction in plaque scores and SM counts with the use of xylitol. But the control group also underwent a similar reduction in SM. This could have been due to the fact that the subjects were not blinded and the control group subjects were aware that they were not receiving xylitol, which may have made them attend to their oral hygiene routines more assiduously than other groups, affecting the outcomes.

In both Akgül *et al*. (15) and Chavan *et al*. (17), chewing gum was provided to both test groups and control groups, which could have biased the results through the mechanical cleaning action of chewing. Nevertheless, in both trials significant reductions in SM were observed in the xylitol groups. In the trial by Chavan *et al*. (17), the control group was not given anything to chew in order to eliminate the antibacterial effect of saliva stimulation by chewing gum, so that any change to SM counts could be attributed to the ingredients of the chewing gum alone.

Kayalvizhi *et al*. (16) investigated the use of cotton swabs for administering xylitol to infants, obtaining a reduction in SM counts that was slightly greater in the xylitol group, although differences did not reach statistical significance. This suggests that the effect is not so much due to xylitol but rather to better oral hygiene practices that clear biofilm. Moreover, the dose of 2.6 g xylitol was less than the recommended daily minimum, which could explain why the expected reduction did not occur.

We observed a similar situation in the trial by Kosara *et al*. (18), who conducted their study among patients presenting poor oral hygiene. Given that both test group and control subjects were asked to brush their teeth twice a day, it is clear that there would be a direct correlation between improvements in oral hygiene and improvements in plaque index, and so it is likely that the reduction observed in SM in both groups was due to improved oral hygiene rather than to xylitol. In any case, the patients were administered 0.02 g xylitol, which is much lower than the dose recommended by the manufacturers.

In the work by Bader *et al*. (19), no significant reduction in the incidence of caries was observed. It should be noted that the placebo was sweetened with sucralose, which could have had a screen effect on xylitol. The trial also differed from most others in that it tested Tablets rather than chewing gum, which eliminated the possible mechanical plaque removal resulting from mastication.

The present literature review came up against several limitations in the search for published articles. In spite of xylitol being an extensively researched topic, and the high number of articles identified in the initial search, after screening abstracts or full texts, and applying the inclusion criteria, fewer works were available for review than had been expected. Most of the articles identified investigated xylitol in combination with some other substance or xylitol compared with some other non-placebo group. Regardless of the potential relevance of these articles, we believe that more studies of the effects of xylitol in comparison with control groups or placebos are needed, particularly in light of the wide disparity of results we observed across the studies reviewed here. Furthermore, although it is well known that the recommended dose of xylitol is 6 g per day, we found that the doses administered in many studies were much lower (16,18-20).

## Conclusions

Based on the results obtained in the studies reviewed, it may be stated that to date, the anticaries effect of xylitol cannot be determined with any certainty, although xylitol may be considered a complimentary agent in caries prevention. The scientific community has not yet developed a standardized protocol to regulate the methods adopted in trials of xylitol, regarding minimum exposure times, the required dosages, and the purity of the xylitol itself. The findings of this systematic review highlight the need to conduct more advanced studies and to standardize the methods used.

## Figures and Tables

**Table 1:** Data extracted from the trials reviewed classified by following variables: study group, age, sex, trial duration, form of xylitol administration, study type.

Authors, Year, Title	Study groups	Age	Sex	Trial duration and dosage	Form and pattern of administration	Study type
Takeuchi et al. (2018) Effects of xylitol-containing chewing gum on the oral microbiota. (12)	76 subjects	Younger or = 20 years	Male	2 days Dosage: 6.78g xylitol daily	(n=39), 2 units xylitol chewing gum 7 times per day for 5 mins (n=37) control group: no chewing gum	Randomized controlled trial
Jain et al. (2022) A Comparative Evaluation of Xylitol Chewing Gum and a Combination of IgY + Xylitol Chewable Tablet on Salivary Streptococcus mutans Count in Children: A Double-blind Randomized Controlled Trial. (13)	120 subjects	6-12 years	Mixed, 61 men and 59 women	15 days Dosage: 1.6g xylitol daily	(n=40) xylitol chewing gum twice a day for 5 mins (n=40) IgY + chewable xylitol Tablet twice a day for 5 minutes (n=40) control group	Double-blinded randomized controlled trial
Masoud et al. (2015) Long-term clinical and bacterial effects of xylitol on patients with fixed orthodontic appliances. (14)	41 subjects	12-30 years with average age of 18.4 years	Mixed	3 months Dosage: 6g xylitol daily	(n=13)2 units xylitol chewing gum 3 times per day for 5 mins (n=13) 4 chewable xylitol tablets 3 times per day (n=12) control group: no xylitol	Pilot clinical trial
Akgül et al. (2020) Effects of short-term xylitol chewing gum on pro-inflammatory cytokines and Streptococcus mutans: A randomized, placebo-controlled trial. (15)	154 subjects	18-65 years, with average age of 23.43 ± 2.3	Mixed, 79 women and 75 men	3 weeks Dosage: 5.4g xylitol daily	(n=77) 2 units xylitol chewing gum 3 times per day for 10 mins (n=77) control group	Double-blinded randomized controlled trial with placebo
Kayalvizhi et al. (2018) Evaluating the Efficacy of Xylitol Wipes on Cariogenic Bacteria in 19- to 35-month-old Children: A Double-blind Randomized Controlled Trial. (16)	44 subjects	19-35 months	Mixed	2 weeks Dosage: 2.6g xylitol daily	(n=22) xylitol towelettes twice a day (n=22) placebo towelettes twice a day	Double-blinded randomized controlled trial
Chavan et al. (2015) Effect of Chewing Xylitol Containing and Herbal Chewing Gums on Salivary Mutans Streptococcus Count among School Children. (17)	72 subjects	12-15 years	Mixed	21 days Dosage:5.14g xylitol daily	(n=24) 1 unit xylitol chewing gum 4 times per day for 10 mins (n=24) herbal chewing gum (n=24) placebo	Randomized clinical trial with follow-up
Koşara et al. (2020) Effects of xylitol impregnated toothbrushes on periodontal status and microbial flora in orthodontic patients. (18)	44 subjects	12-18 years	Mixed, 22 men and 22 women	3 months Dosage: 0.02g xylitol daily	(n=22) brushing with xylitol twice a day for 2 mins (n=22) brushing without xylitol	Clinical study with parallel groups
Bader et al. (2013) Results from the Xylitol for Adult Caries Trial (X-ACT). (19)	691 subjects	21-80 years	Mixed	33 months Dosage: 5g xylitol daily	(n=344) 1 xylitol Tablet 5 times per day (n=347) placebo group	Double-blinded randomized controlled trial with placebo
Mäkinen et al. (2013) Topical xylitol administration by parents for the promotion of oral health in infants: a caries prevention experiment at a Finnish Public Health Centre. (20)	271 subjects	6-8 months	Mixed	26-28 months Dosage: 13.5mg daily per deciduous tooth	(n=133) xylitol applied with cotton bud twice a day (n=138) control group	Clinical trial

## Data Availability

The datasets used and/or analyzed during the current study are available from the corresponding author.
